# Raman Evidence of Moiré Diamondene Formation by High‐Pressure

**DOI:** 10.1002/advs.76467

**Published:** 2026-07-09

**Authors:** Chaofeng Gao, Mingming Chang, Mengting Wang, Yu Chen, Shiyun Zheng, Jiankuan Wu, Min Chen, Keying Han, Luyuan Fan, Zhenxiao Zhang, Kun Zhai, Bochong Wang, Congpu Mu, Yingchun Cheng

**Affiliations:** ^1^ State Key Laboratory of Metastable Materials Science & Technology and Key Laboratory of Microstructural Material Physics of Hebei Province School of Science Yanshan University Qinhuangdao P. R. China; ^2^ State Key Laboratory of Flexible Electronics (LoFE) & Institute of Advanced Materials (IAM) School of Flexible Electronics (Future Technologies) Nanjing Tech University (Nanjing Tech) Nanjing P. R. China

**Keywords:** high pressure, moiré diamondene, Raman spectroscopy, twisted bilayer graphene

## Abstract

Moiré diamondene, a two‐dimensional diamond‐like material with partial interlayer covalent bonding, is predicted to host strongly correlated quantum effects but has not yet been experimentally synthesized. It exhibits a hybrid sp^2^–sp^3^ bonding character, comprising sp^2^‐hybridized graphene domains and sp^3^‐hybridized nano‐diamond domains. We predict that moiré diamondene can be formed by applying a pressure of approximately 2 GPa to twisted bilayer graphene (TBG) on a diamond surface by first‐principles calculations. Under a pressure of 2 GPa, the experimental Raman G band of TBG on the diamond anvil culet splits into two distinct peaks, denoted as the N1 and N2 bands. Supported by comparative experiments and theoretical modeling, we attribute the N1 band to the interlayer bonded regions and the N2 band to the unbonded graphene segments. Therefore, the G band splitting serves as direct spectroscopic evidence for the formation of moiré diamondene. We demonstrate that the synthesis relies on the simultaneous presence of high pressure, an interlayer twist angle, and a chemically active diamond (100) surface, which paves the way for synthesizing moiré diamondene.

## Graphical abstract

Two‐dimensional moiré diamondene is formed at approximately 2 GPa through the synergy of high pressure, interlayer twist, and a chemically active diamond (100) surface. It is a hybrid sp^2^–sp^3^ nanostructure, consists of localized nanodiamond domains and unbonded graphene segments. The Raman G band splitting serves as direct spectroscopic evidence for the formation of two‐dimensional moiré diamondene.

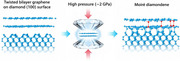

## 1 Introduction

The discovery of correlated insulating states [1] and superconductivity [2] in magic‐angle twisted bilayer graphene (TBG) has significantly advanced the study of strongly correlated electronic states in moiré superlattices [3, 4, 5, 6, 7, 8]. Flat bands emerge only at minimal twist angles in TBG, leading to a narrow magic‐angle window [9, 10]. Theoretical studies predict that moiré diamondene with interlayer covalent bonds exhibits a wide band gap and flat bands at large twist angles, which overcomes the limitation of the narrow magic‐angle window of conventional TBG [11, 12, 13]. Notably, the electronic states of the flat bands in moiré diamondene exhibit spatial distributions similar to triangular and Kagome lattices [14]. Therefore, moiré diamondene has emerged as an ideal platform for exploring novel strongly correlated quantum effects, including moiré excitons, Mott insulators, and zero‐magnetic‐field fractional quantum Hall states [15, 16, 17]. However, the controlled synthesis of moiré diamondene remains elusive, hindering both fundamental research and potential applications.

The fabrication of moiré diamondene relies on the formation of interlayer sp^3^ covalent bonds within TBG [18, 19]. Fortunately, well‐developed synthetic methods for untwisted 2D diamond‐related materials offer crucial insights into overcoming this challenge [12, 20, 21, 22, 23]. For example, Barboza et al. pioneered the synthesis of 2D diamond‐like crystals under high pressure in a hydroxylated atmosphere [24]. By inducing out‐of‐plane strain through nanoindentation, a diamond‐like carbon film with stiffness and hardness comparable to bulk diamond has been prepared [25]. Additionally, 2D diamondene has been successfully synthesized under a pressure of 2 GPa using hydroxyl functionalization in water combined with diamond anvil cell (DAC) technology [26]. The synthesis of pure diamond thin films at pressures above 20 GPa without chemical modification is also reported [27]. More recently, it is reported that the high‐pressure‐induced structural phase transition of hexagonal boron nitride can effectively promote interlayer bonding in graphene [28]. These studies indicate that high pressure is an effective method for generating interlayer sp^3^ covalent bonds in graphene [29]. Combining high pressure with chemical functionalization can optimize the reaction pathway [30, 31, 32, 33], providing a promising route for the controllable synthesis of 2D moiré diamondene.

In this study, we first predict the formation of moiré diamondene from TBG under high pressure by means of first‐principles calculations. Guided by this prediction, we investigate the high‐pressure spectral evolution of TBG using in situ Raman spectroscopy. We observe that the Raman G band of TBG on the diamond anvil culet splits into two new peaks (N1 and N2 bands) at 2 GPa, which provides direct spectroscopic evidence for the phase transition. Comparative experiments confirm that the formation of moiré diamondene originates from the combined effects of high pressure, the diamond anvil culet, and the twist angle. Subsequently, theoretical calculations elucidate the origin of the observed characteristic Raman peaks. These findings not only provide an effective pathway for the synthesis of 2D moiré diamondene but also establish a solid foundation for future experimental studies.

## 2 Results and Discussion

**FIGURE 1:**
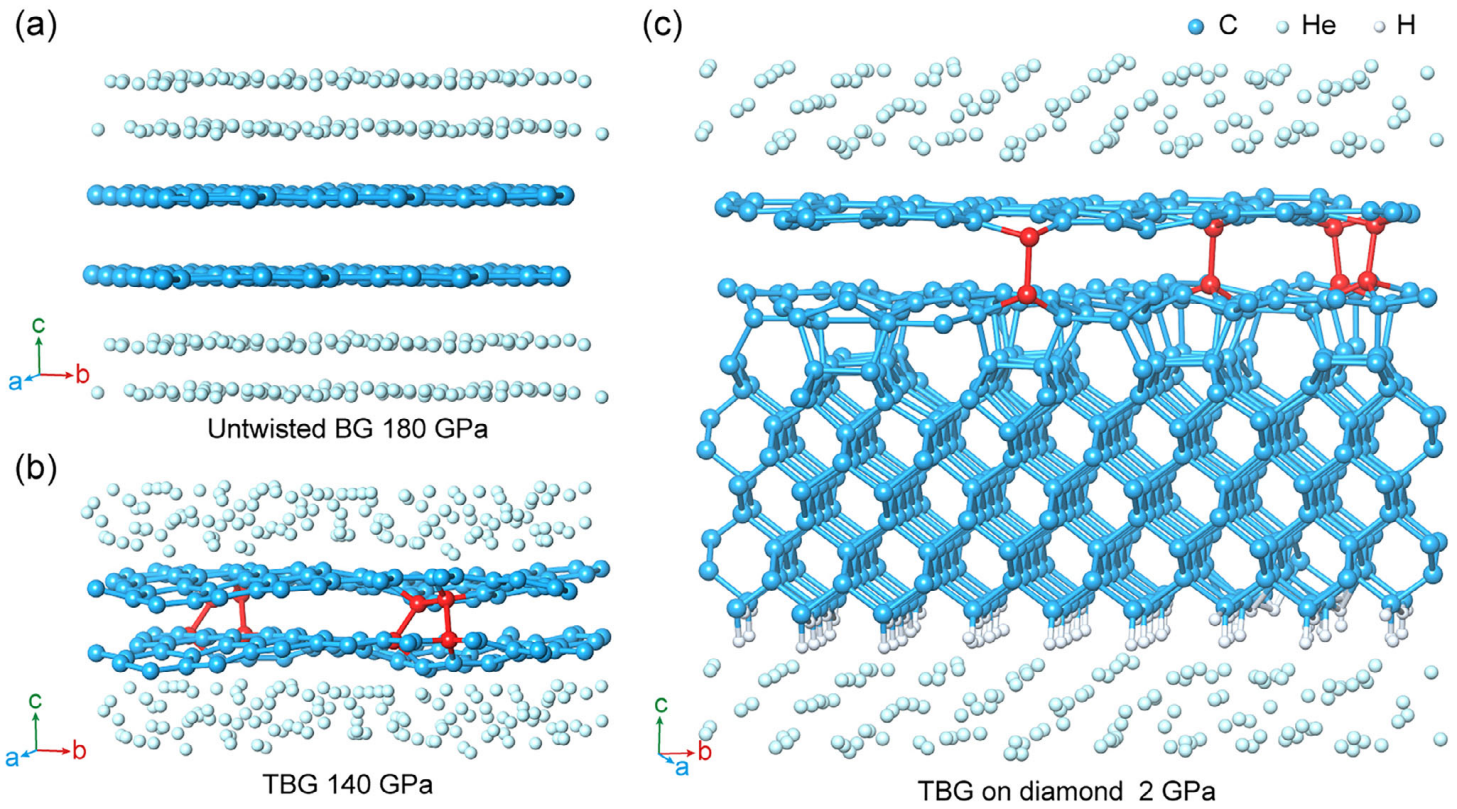
Structural models for moiré diamondene formation. (a) Atomic structure of untwisted BG at 180 GPa. (b) The 13.2° TBG at 140 GPa. (c) The 13.2° TBG on a diamond (100) surface at 2 GPa. Chemically inert He atoms serve as the pressure‐transmitting medium.

To verify the feasibility of moiré diamondene formation, we investigate the atomic structural evolution of TBG on the diamond (100) surface under high pressure by means of first‐principles calculations. For comparison, we first examine the structural evolution of untwisted BG and 13.2° TBG under pressure ranging from 0 to 180 GPa (Figures S2 and S3). The interlayer spacing of untwisted BG gradually decreases, reaching 2.0 Å at 180 GPa (Figure 1a). The shortest interlayer C‐C distance is 2.05 Å, which is much longer than the C‐C bond length (1.54 Å) in cubic or hexagonal diamond [34]. In contrast, ripples gradually emerge in the 13.2° TBG as the pressure increases. Interlayer rotation in TBG leads to the formation of localized AA‐, AB‐, and SP‐stacked regions within the moiré superlattice. At 140 GPa, interlayer bonding forms in the 13.2° TBG, with some interlayer C‐C bonds having a length of 1.6 Å (Figure 1b), indicating the formation of sp^3^ C─C bonds. The interlayer sp^3^ C─C bonds in 13.2° TBG are primarily located in the AA‐stacked regions (Figure S3) due to the lower formation energy [19, 35]. Although interlayer twisting effectively promotes interlayer bonding in BG, the critical pressure of 140 GPa is too high.

We next explore the influence of the diamond (100) surface on the interlayer bonding in TBG (Figure 1c and Figure S4). Under high pressure, carbon atoms in the bottom layer of 13.2° TBG form bonds with the carbon atoms on the active dimer diamond (100) surface. This results in the activation of carbon atoms in the bottom layer of 13.2° TBG. These activated carbon atoms in the bottom layer further bond with carbon atoms in the top layer of TBG. As a consequence, interlayer sp^3^‐hybridized covalent C─C bonds form between the adjacent graphene layers, leading to the formation of moiré diamondene. Most interlayer‐bonded atoms are located within the AA‐stacked regions of the TBG (Figure S4). The presence of the diamond (100) surface substantially reduces the critical pressure for interlayer C‐C bonding formation in TBG, enabling the formation of moiré diamondene at 2 GPa, which is characterized by localized sp^3^‐hybridized nanodiamond islands.

**FIGURE 2:**
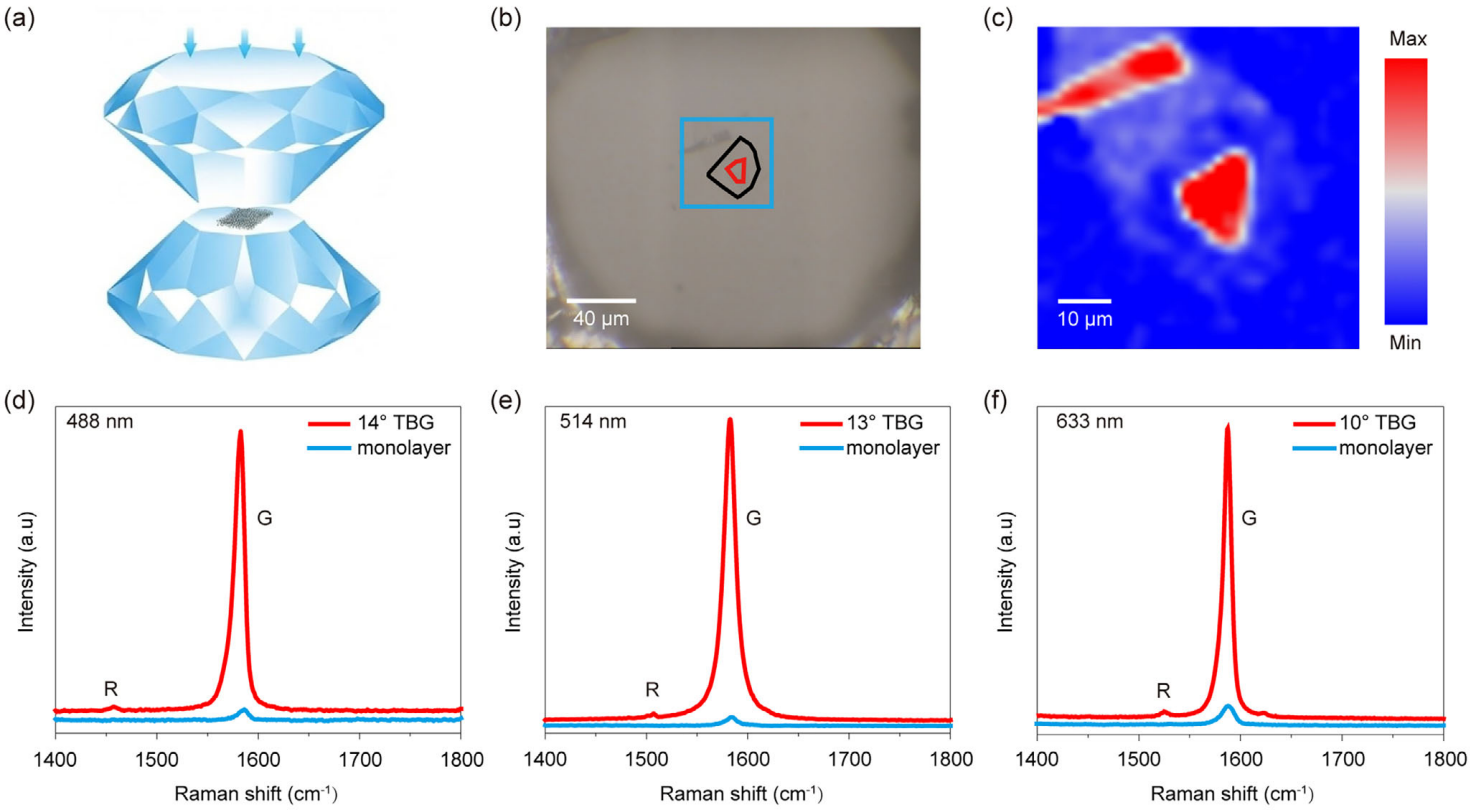
Raman characterization of TBG samples under ambient condition. (a) Schematic illustration of TBG in a DAC. (b) Optical micrograph of TBG on diamond anvil culet of DAC. Red and black outlines indicate the TBG and monolayer graphene, respectively. (c) Raman intensity mapping of the G band for the region enclosed by the blue box in (b). (d–f) Raman spectra of bilayer graphene with twist angle of 14°, 13°, and 10° on diamond anvil culet. Raman spectra of monolayer graphene are included for comparison.

To validate the above theoretical prediction, we apply high pressure to BG or TBG using a DAC. We take TBG on diamond as an example to illustrate sample preparation and characterization (Figure 2a). In this configuration, the TBG is firmly attached to the diamond (100) surface. Due to the high optical transparency of both graphene and diamond, it is difficult to distinguish 14° TBG from the diamond anvil by optical microscopy (Figure 2b). Figure 2c displays the G band intensity mapping of graphene within the blue‐boxed region of Figure 2b, obtained using an excitation wavelength of 488 nm. The bright red area in the center corresponds to the TBG, and the bright red region in the top‐left corner represents the few‐layer graphene.

The Raman spectra of 14° TBG and monolayer graphene, acquired with an excitation wavelength of 488 nm, are presented in Figure 2d. The G band intensity ratio between 14° TBG and monolayer graphene reaches 25.3, indicating a pronounced enhancement attributed to a resonance effect arising from the selective absorption at specific excitation wavelengths [36, 37]. Additionally, a twist‐induced R band appears at a frequency of 1457.6 cm^−1^. The R band arises from the intravalley electron transitions in TBG and monotonically red‐shifts with increasing twist angle [38, 39, 40]. Since the excitation wavelength for G band enhancement and the position of the R band are related to the twist‐angle, the twist angle of the as‐prepared TBG sample can be determined. Based on the comprehensive analysis of these two spectral features, the twist angle of the corresponding TBG sample in Figure 2b is confirmed to be approximately 14°.

Additionally, we also fabricate TBG samples with twist angles of 13° and 10°. To confirm these twist angles, we measure the Raman spectra of as‐prepared TBG samples (Figure 2e,f). The Raman spectra for the 13° and 10° TBG samples are collected at excitation wavelengths of 514 and 633 nm, respectively. Similarly, the G band intensities of the 13° and 10° TBG samples also exhibit significant enhancement relative to that of monolayer graphene, with intensity ratios of 35.8 and 15.85, respectively. The frequencies of R band are 1506.6 and 1525.4 cm^−1^ for the 13° and 10° TBG samples, respectively. The Raman characterizations confirm the twist angles of the prepared TBG samples and demonstrate their excellent quality.

**FIGURE 3:**
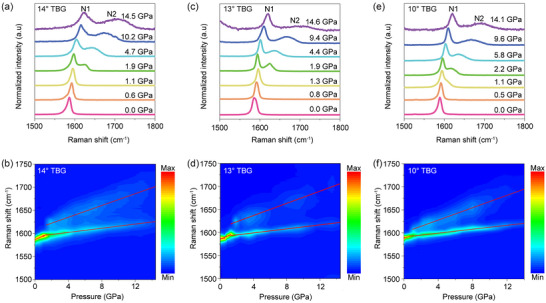
Pressure‐dependent Raman evolution of TBG samples on diamond anvil culet. Normalized Raman spectra and intensity contour maps for (a, b) 14°, (c, d) 13°, and (e, f) 10° TBG under pressure up to 14 GPa, respectively. As pressure increases, two new peaks (N1 and N2) emerge around 1600 cm^−1^. The raw Raman spectra are displayed in Figure S5.

Subsequently, we focus on the Raman spectra of TBG samples firmly attached to the diamond anvil culet under high pressure. As shown in Figure 3a,b, the G band of the 14° TBG sample exhibits a pronounced blue‐shift with increasing pressure. At 1.9 GPa, the G band of 14° TBG splits into two new peaks, labeled N1 and N2, located at 1596.6 and 1623.1 cm^−1^, respectively. With further increase in pressure, both peaks continually undergo a blue‐shift. The slopes of frequency versus pressure for G, N1, and N2 bands of 14° TBG are 8.2, 2.2, and 6.4 cm^−1^GPa^−1^, respectively, as listed in Table S1. The corresponding linear fits to the Raman shift data are shown in Figure S6.

Similarly, the high‐pressure Raman spectra of the 13° and 10° TBG samples also exhibit two new peaks near 1600 cm^−1^, appearing at 1.9 and 1.1 GPa, respectively (Figure 3c–e). For the 13° TBG sample, the N1 and N2 peaks are located at 1594.0 and 1626.7 cm^−1^, while for the 10° TBG sample, the peaks are found at 1593.1 and 1608.1 cm^−1^. As pressure increases, these peaks also undergo a blue‐shift. For the 13° TBG sample, the slopes of the N1 and N2 bands are 2.2 and 6.6 cm^−1^GPa^−1^, respectively, while for the 10° TBG sample, the shift rates are 2.1 and 6.7 cm^−1^GPa^−1^.

For all three TBG samples on the diamond anvil culet, two new Raman peaks emerge near 1600 cm^−1^ under high pressure, with a critical pressure of approximately 2 GPa. Such changes in the Raman spectrum typically signal a structural phase transition under high pressure [41, 42, 43]. Based on the theoretical prediction of moiré diamondene formation from TBG on a diamond surface under high pressure (Figure 1c) and the experimental observation of G band splitting, we assume the N1 and N2 peaks are attributed to sp^2^‐ and sp^3^‐hybridization in moiré diamondene.

**FIGURE 4:**
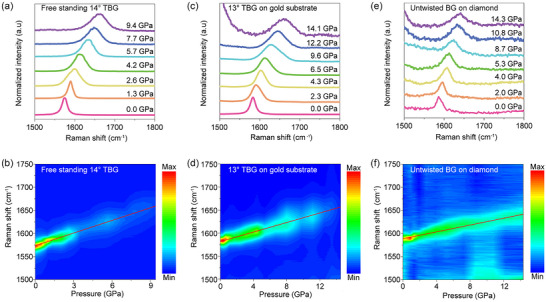
High‐pressure Raman response of free‐standing 14° TBG, 13° TBG on a gold substrate, and untwisted BG on a diamond anvil culet. (a) Normalized Raman spectra of free‐standing 14° TBG at pressures ranging from 1 atm to 9.4 GPa. (b) Corresponding Raman intensity contour map of free‐standing 14° TBG. Raman spectra and corresponding intensity maps of (c, d) 13° TBG on gold substrate and (e, f) untwisted BG on diamond anvil culet. Pressures for 13° TBG and untwisted BG range from 1 atm to ≈14 GPa, respectively. The raw Raman spectra are displayed in Figure S7.

To verify the assumption of the origin of N1 and N2 peaks, we then study the high‐pressure Raman response of free‐standing 14° TBG, 13° TBG on a gold substrate, and untwisted BG on a diamond anvil culet for comparison. For the free‐standing 14° TBG under pressure from 1 atm to 9.4 GPa, only a single G peak is observed in the range of 1500 to 1750 cm^−1^ (Figure 4a,b). The Raman spectra of 13° TBG on a gold substrate and untwisted BG on a diamond anvil culet under pressure are similar to that of free‐standing TBG (Figure 4c–f). The G bands of these samples all exhibit smooth blue‐shift under high pressure with slopes of 9.3, 5.4, and 3.7 cm^−1^GPa^−1^, respectively (Table S1 and Figure S8). We note that the G band does not split under high pressure for free‐standing TBG, TBG on gold substrate, or untwisted BG on a diamond anvil culet, indicating the absence of diamond formation. Therefore, N1 and N2 peaks in TBG on diamond anvil culet under high pressure are highly attributed to the formation of moiré diamondene.

**FIGURE 5:**
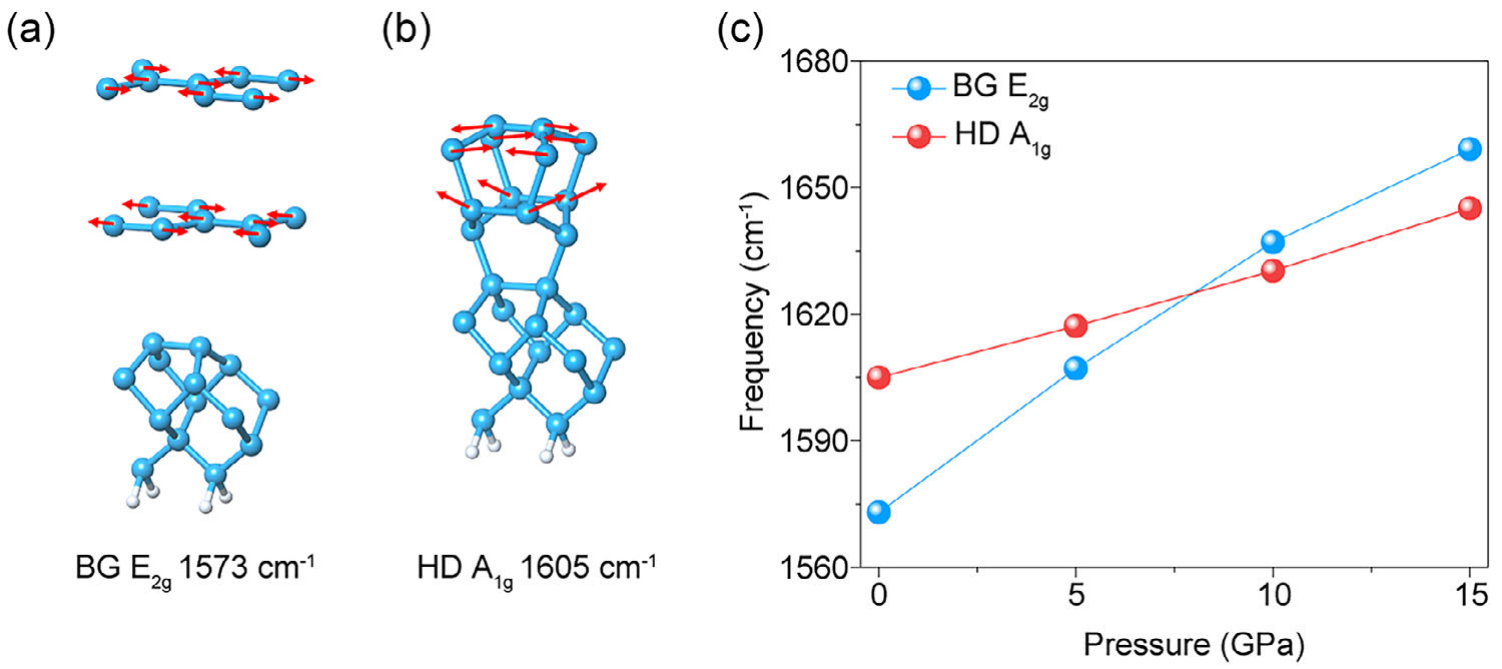
Calculated phonon properties of simplified models for moiré diamondene. (a) Schematic of the atomic displacements of the E_2g_ phonon mode for BG on a diamond (100) surface. (b) Schematic of the atomic displacements of the A_1g_ phonon mode for hexagonal diamond (HD) bonded to a diamond (100) surface. (c) Calculated frequency evolution of the E_2g_ and A_1g_ modes as a function of pressure up to 15 GPa.

To verify the above assumption, we calculate the phonon properties of bilayer graphene and diamondene under high pressure. The structure of compressed 13.2° TBG on the diamond (100) surface (shown in Figure 1c) consists of 418 atoms, which is too large for phonon frequency calculations based on density functional theory methods. Therefore, we employ simplified structural models to mimic moiré diamondene for the phonon calculations. For the interlayer unbonded regions of moiré diamondene on the diamond anvil culet, an untwisted BG/diamond (100) surface structure is utilized as the simplified model. In contrast, for the interlayer bonded nano‐diamond islands regions, a hexagonal diamond/diamond (100) surface configuration is adopted as the simulation structure. The phonon frequency calculations demonstrate that the frequency of the G band related E_2g_ mode of untwisted BG on the diamond (100) surface is 1573 cm^−1^ at ambient pressure (Figure 5a). For the hexagonal diamond bonded to the diamond (100) surface, the A_1g_ mode exhibits a vibrational frequency of 1605 cm^−1^ (Figure 5b). The calculated frequencies for both the E_2g_ mode of BG and the A_1g_ mode of hexagonal diamond on the diamond (100) surface are around 1600 cm^−1^, consistent with the experimentally observed frequencies of the N1 and N2 bands at 2 GPa.

We also calculate the phonon frequencies of the E_2g_ mode of BG and the A_1g_ mode of hexagonal diamond under pressures up to 15 GPa (Figure 5c). Both the graphene E_2g_ and the hexagonal diamond A_1g_ modes on the diamond (100) surface exhibit characteristic blue‐shifts with increasing pressure. Notably, the E_2g_ mode exhibits a significantly larger pressure coefficient (5.8 cm^−1^GPa^−1^) than the A_1g_ mode (2.7 cm^−1^GPa^−1^). Moreover, the frequency of the E_2g_ mode exceeds that of the A_1g_ mode as pressure increases. This theoretical prediction is consistent with our experimental observations, where the N2 band exhibits a significantly steeper slope (6.7 cm^−1^GPa^−1^) compared to the N1 band (2.2 cm^−1^GPa^−1^) and is located at a higher frequency. Based on these distinct features, we assign the N1 band to the A_1g_ mode of the interlayer bonded nano‐diamond islands in moiré diamondene, and the N2 band corresponds to the E_2g_ mode of the unbonded graphene regions.

While theoretical calculations attribute the G‐band splitting to moiré diamondene, other possible origins should be excluded. One possible alternative is conventional high‐pressure‐induced structural changes, including partial amorphization and M‐carbon formation. Partial amorphization of few‐layer graphene occurs above ≈16 GPa and gives rise to broad D and G bands near 1350 and 1580 cm^−1^, respectively [44, 45, 46]. M‐carbon, a bulk sp^3^‐bonded phase of cold‐compressed graphite, forms above ≈17 GPa [47, 48] and exhibits a broadened, weakened G band. By contrast, the N1 and N2 bands emerge at ≈2 GPa and appear as two well‐resolved peaks near 1600 cm^−1^, excluding partial amorphization and M‐carbon formation as their origin. Uniaxial strain can also be excluded, because uniaxial strain‐induced G band splitting produces two symmetric peaks [49, 50], whereas the N1 and N2 bands exhibit different intensities and full‐width at half‐maximum.

## 3 Conclusions

In summary, we have investigated the high‐pressure formation of 2D moiré diamondene on a diamond anvil culet. Direct spectroscopic evidence for the formation of 2D moiré diamondene is obtained by combining high‐pressure diamond anvil cell techniques with Raman spectroscopy. Specifically, the Raman N1 and N2 bands associated with 2D moiré diamondene emerge near 1600 cm^−1^ at a pressure of approximately 2 GPa. Our experimental results further reveal that the formation of 2D moiré diamondene arises from the synergistic effects of high pressure, interlayer twist, and the catalytic role of the diamond anvil culet. Additionally, first‐principles calculations elucidate the atomic structural transformation from TBG to moiré diamondene, providing a detailed understanding of the origin of the experimental N1 and N2 bands. The 2D moiré diamondene features localized interlayer bonding, where the N1 and N2 bands correspond to the interlayer bonded nano‐diamond islands and unbonded graphene domains, respectively. Our work thus establishes a viable pathway for synthesizing 2D moiré diamondene and lays the foundation for future experimental studies of this novel material.

## Author Contributions

**K.Z**., **B.C.W**., **C.P.M**., and **Y.C.C**. supervised the project. **C.F.G**., **M.M.C**., **M.T.W**., **Y.C**., **S.Y.Z**., **J.K.W**., **M.C**., **K.Y.H**., **L.Y.F**., and **Z.X.Z**. performed the experiments, **C.F.G**. carried the calculations. **C.F.G**., **M.M.C**., and **Y.C**. analyzed the data and drafted the manuscript, and all authors provided input on the manuscript. All authors discussed the results and made contributions to the manuscript.

## Conflicts of Interest

The authors declare no conflicts of interest.

## Supporting information




**Supporting File**: advs76467‐sup‐0001‐SuppMat.docx.

## Data Availability

The data that support the findings of this study are available from the corresponding author on reasonable request.
