# Supplementary material for: Raman Evidence of Moiré Diamondene Formation by High‐Pressure
Source: Adv Sci (Weinh). 2026 Jul 9:e76467. Online ahead of print. doi: 10.1002/advs.76467 (PMC13348332; doi:10.1002/advs.76467)
Supplement: Supplementary file 1 — Supporting File: advs76467‐sup‐0001‐SuppMat.docx. [file ADVS-9999-e76467-s001.docx]

Supporting Information for

**Raman Evidence of Moiré Diamondene Formation by High-Pressure**

Chaofeng Gao,^1^ Mingming Chang,^1^ Mengting Wang,^1^ Yu Chen,^1^ Shiyun Zheng,^1^ Jiankuan Wu,^1^ Min Chen,^1^ Keying Han,^1^ Luyuan Fan,^1^ Zhenxiao Zhang,^2^ Kun Zhai,^1^ Bochong Wang,^1^ Congpu Mu,^1^ and Yingchun Cheng^1,*^

^1^ State Key Laboratory of Metastable Materials Science & Technology and Key Laboratory of Microstructural Material Physics of Hebei Province, School of Science, Yanshan University, Qinhuangdao, 066004, P. R. China

^2^ State Key Laboratory of Flexible Electronics (LoFE) & Institute of Advanced Materials (IAM), School of Flexible Electronics (Future Technologies), Nanjing Tech

University (Nanjing Tech), 5 Xinmofan Road, Nanjing, 210009, P. R. China

**^*^Author to whom correspondence should be addressed:**

iamyccheng@ysu.edu.cn;

**Keywords:** twisted bilayer graphene, high pressure, moiré diamondene, Raman spectroscopy.

**Outline**

[1. Methods 1](#_Toc28218)

[1.1 Calculation details 1](#_Toc22951)

[1.2 Sample preparation 1](#_Toc22518)

[1.3 High-pressure Raman measurements 2](#_Toc14833)

[2. Theoretical structural evolution under high pressure 2](#_Toc24889)

[3. Detailed high-pressure Raman characterization results 4](#_Toc6123)

**1. Methods**

**1.1 Calculation details**

All our calculations in this work are performed by using the PWmat code [1], which is based on density functional theory using a plane-wave basis set. The generalized gradient approximation with the Perdew-Burke-Ernzerhof parametrization [2] is adopted. The structural optimization is continued until the residual forces have converged to less than 0.001 eV/Å. A cutoff energy of 50 Ry and a 1×1×1 k-point grid are employed. The van der Waals interlayer interaction is corrected by using the DFT-D3 functional [3]. Phonon calculation is carried out using the phonopy program [4], with force constants derived from the PWmat code. For the force constant calculations, a 2×1×1 unit cell with a 12×12×1 *k*-point mesh is used.

**1.2 Sample preparation**

Graphene flakes are prepared on silicon substrates by micromechanical exfoliation. The silicon substrates are pre-cleaned with 100 mW oxygen plasma for 3 minutes to enhance the yield of monolayer graphene. **Figure S1** presents the optical image and Raman spectra of the exfoliated monolayer and bilayer graphene. For monolayer graphene, the 2D band appears sharp and symmetric with an ideal Lorentzian line shape, where the intensity of the 2D band exceeds that of the G band. In contrast, the 2D band of bilayer graphene is resolved into four distinct Lorentzian peaks, and its intensity is weaker than that of the G band. These typical Raman features confirm the monolayer and bilayer nature of the exfoliated graphene [5]. Twisted bilayer graphene (TBG) samples were prepared by a tear-and-stack dry-transfer method using a PPC/PDMS transfer film [6-7]. Part of a monolayer graphene flake was first picked up by the transfer film, leaving the residual graphene on the SiO_2_/Si substrate. The substrate containing the residual graphene was rotated by a preset nominal angle. The picked-up graphene and residual graphene were then aligned and stacked together to form TBG with the target twist angle. Finally, the TBG samples were transferred onto the diamond anvil culet, gold substrates, and gold grids for high-pressure Raman measurements.


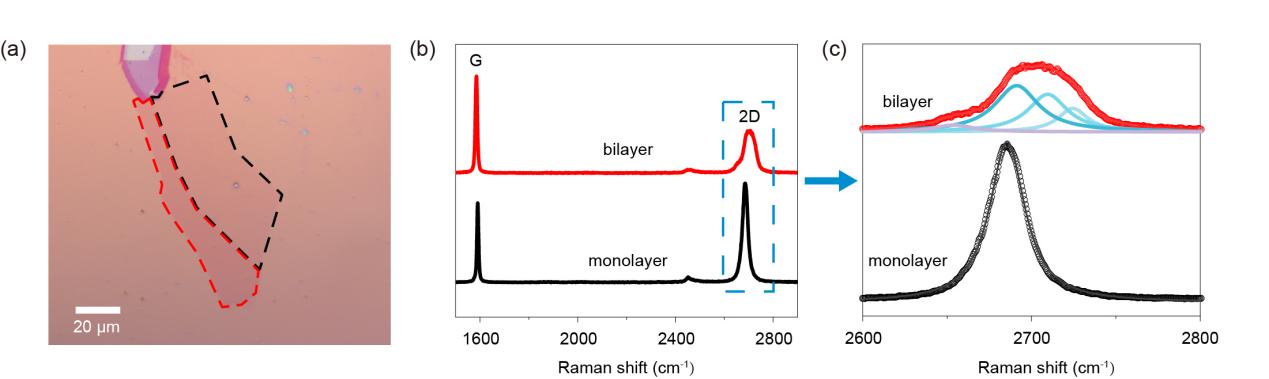


**Figure S1.** (a) Typical optical image of monolayer and bilayer graphene on a Si/SiO_2_ substrate. (b) Raman spectra of monolayer and bilayer graphene. (c) Enlarged view of the 2D bands shown in (b).

**1.3 High-pressure Raman measurements**

A diamond anvil cell is used to apply high pressure on the TBG, with silicone oil serving as the pressure-transmitting medium and a 10 μm ruby ball as the pressure calibrant [8-9]. A T301 steel gasket with a 200 μm diameter hole is used as the sample chamber. Raman characterization is performed using a WITec alpha300R confocal micro-Raman system with excitation laser wavelengths of 488, 514, and 633 nm. The spectra were acquired using an 1800 grooves/mm grating, yielding a spectral resolution of ~1 cm^-1^. The peak frequencies were determined by spectral fitting. The laser power on the sample is kept below 0.5 mW to avoid laser-induced heating or damage. The acquisition time for each Raman spectrum is 200 s. All high-pressure Raman measurements are carried out at room temperature. A long working distance objective lens (Olympus, LMPLN20x) is employed for the high-pressure Raman measurements.

**2. Theoretical structural evolution under high pressure**


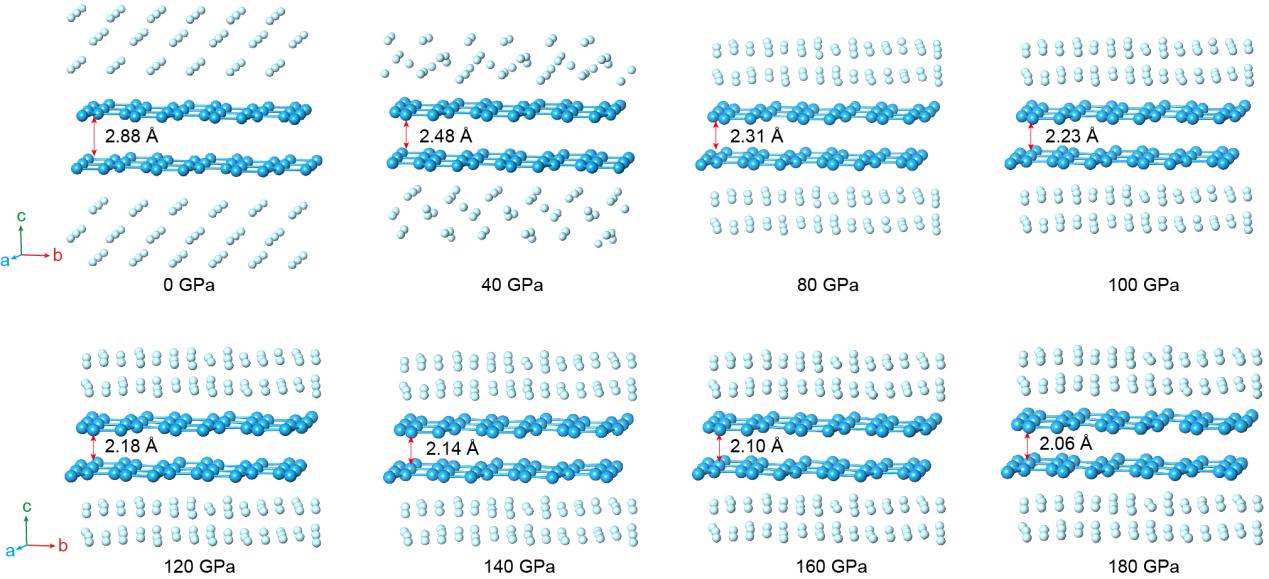


**Figure S2.** Theoretical structural evolution of untwisted bilayer graphene under pressures ranging from 0 to 180 GPa.


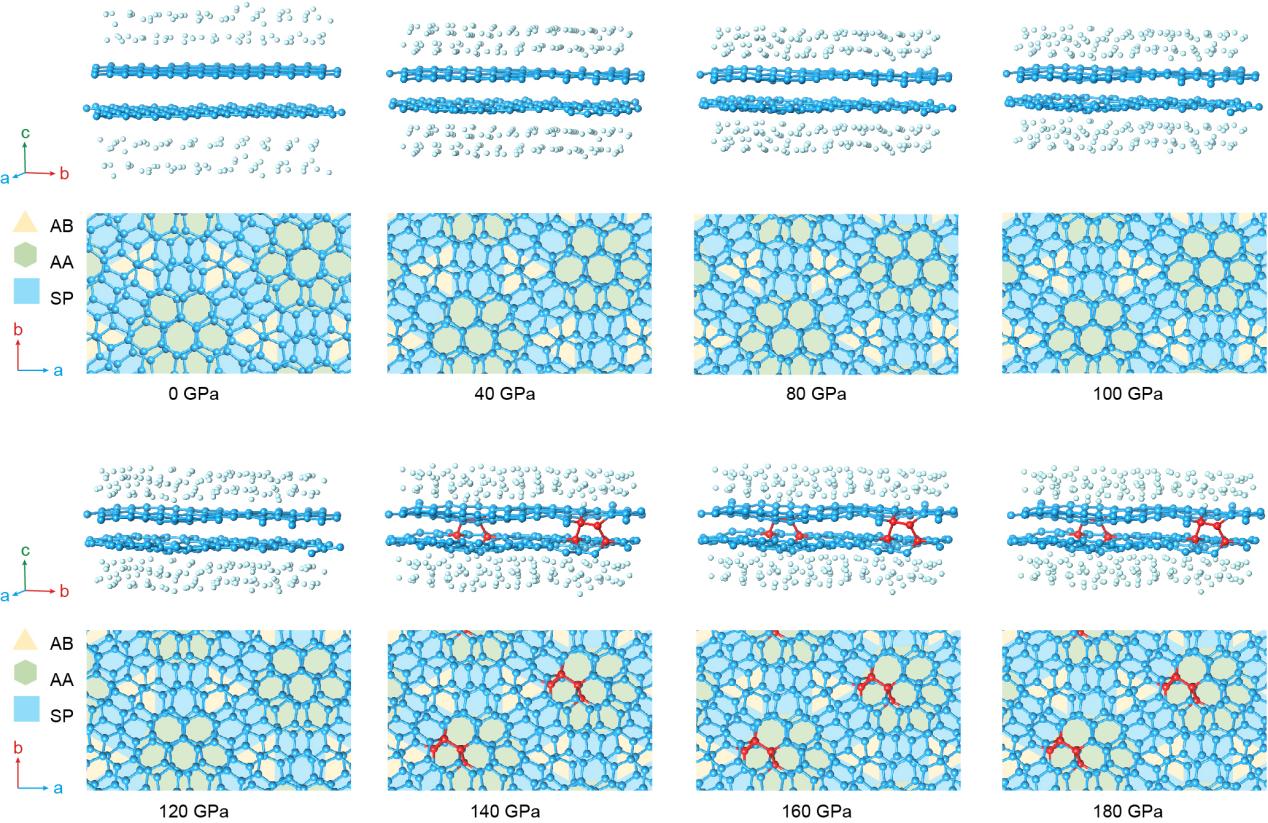


**Figure S3.** Theoretical structural evolution of 13.2° TBG as pressure increases from 0  to 180 GPa.


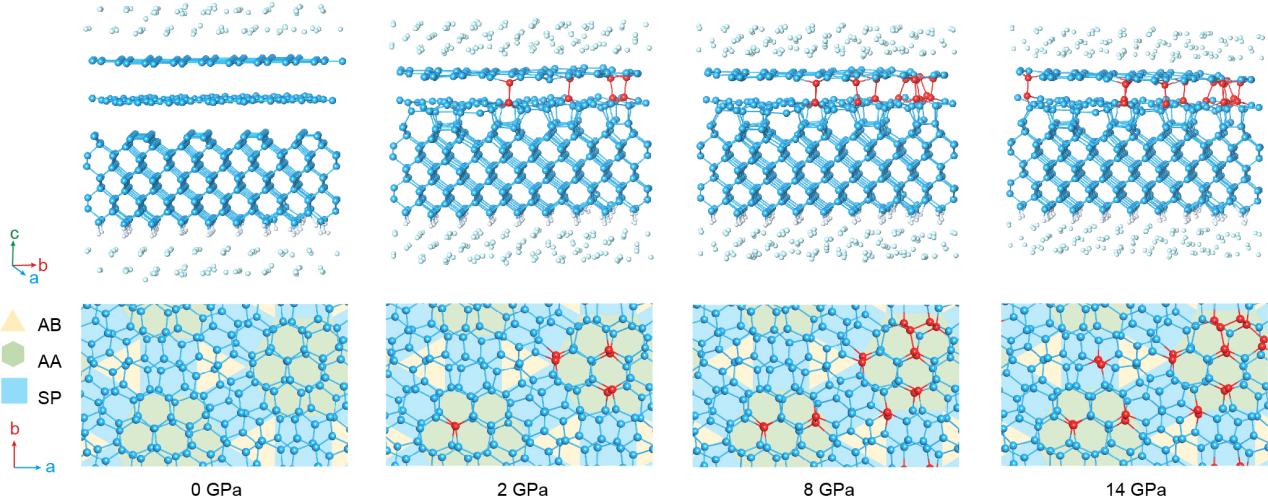


**Figure S4.** Calculated structural evolution of 13.2° TBG on diamond anvil under compression ranging from 0 to 180 GPa.

**3. Detailed high-pressure Raman characterization results**


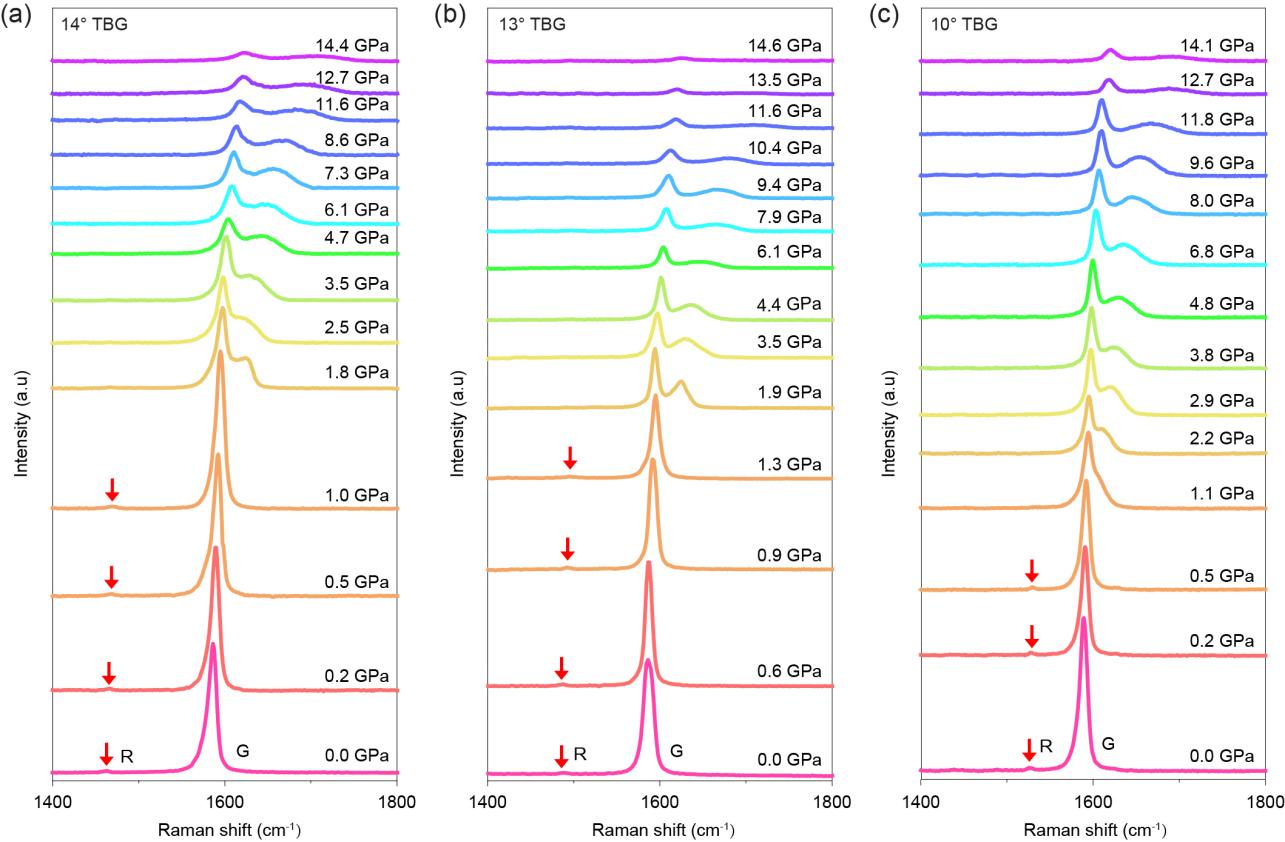


**Figure S5.** Raw pressure-dependent Raman evolution of (a) 14°, (b) 13°, and (c) 10° TBG on diamond anvil culet, with the weak R band highlighted by red arrows.


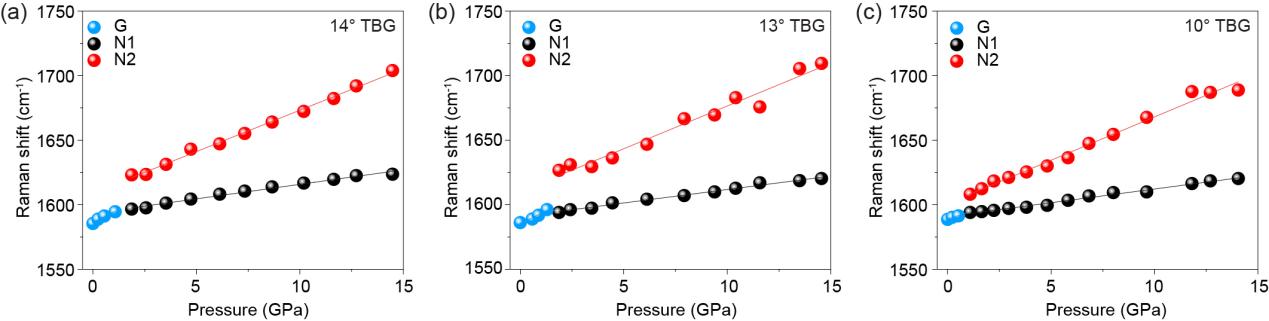


**Figure S6.** Raman shifts of G, N1, and N2 bands for (a) 14°, (b) 13°, and (c) 10° TBG on diamond anvil culet as a function of pressure.


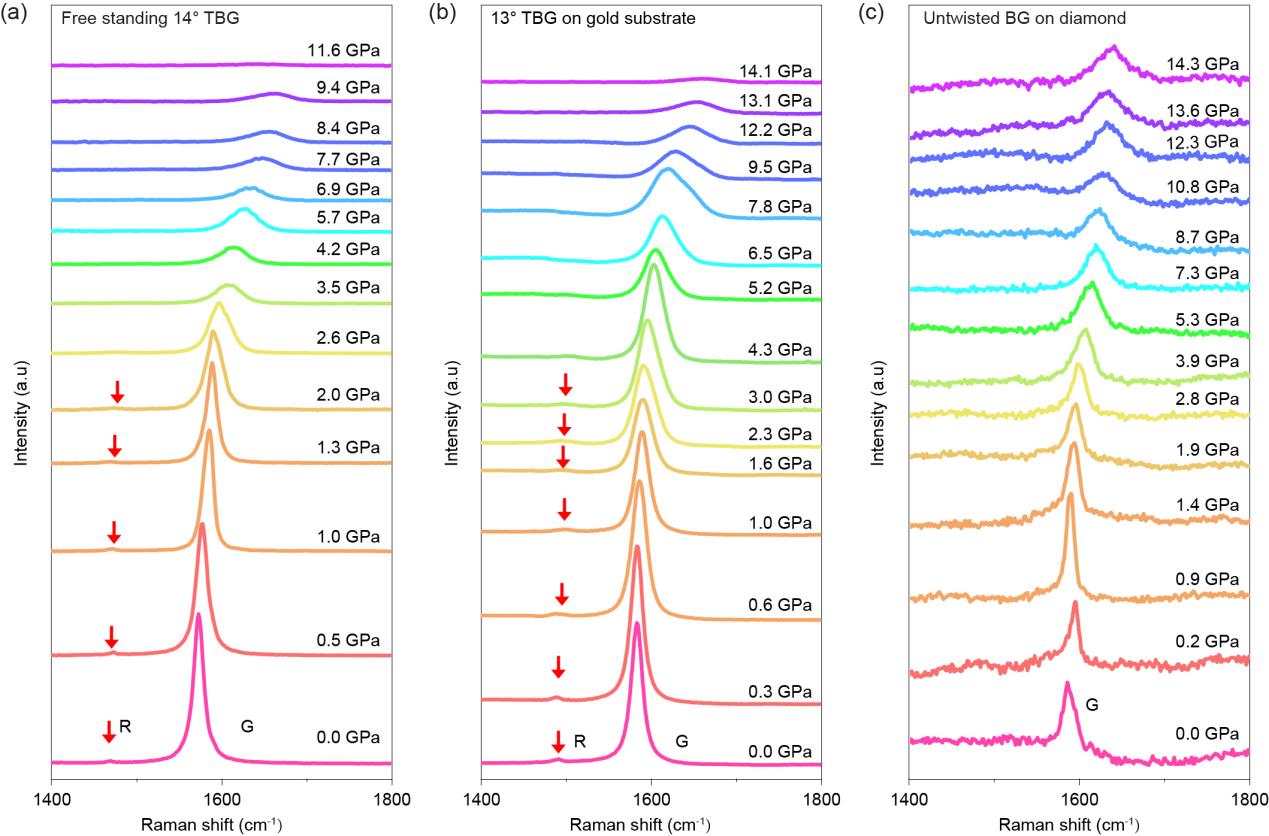


**Figure S7.** Raw pressure-dependent Raman evolution of (a) free-standing 14° TBG, (b) 13° TBG on gold substrate, and (c) untwisted BG on diamond anvil culet, with the weak R band highlighted by red arrows.


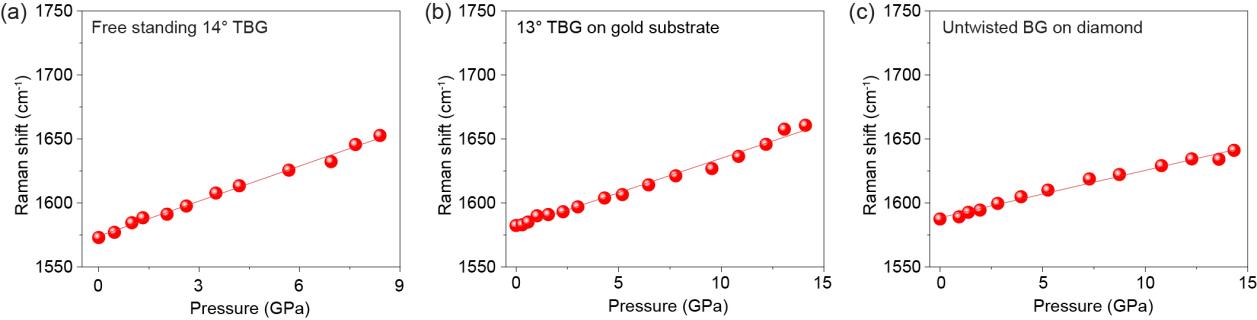


**Figure S8.** Pressure dependence of the Raman G band frequencies for (a) free standing 14° TBG, (b) 13° TBG on gold substrate, and (c) untwisted BG on diamond anvil culet.

Table S1 | Shift rates of Raman peaks of graphene under pressure (cm^-1^GPa^-1^).

|  | G | N1 | N2 |
| --- | --- | --- | --- |
| 14° TBG on diamond anvil culet | 8.2±0.6 | 2.2±0.1 | 6.4±0.1 |
| 13° TBG on diamond anvil culet | 7.8±1.1 | 2.2±0.1 | 6.6±0.4 |
| 10° TBG on diamond anvil culet | 5.2±0.8 | 2.1±0.1 | 6.7±0.2 |
| Free standing 14° TBG | 9.3±0.5 | - | - |
| 13° TBG on gold substrate | 5.4±0.1 | - | - |
| BG on diamond anvil culet | 3.7±0.1 | - | - |

**REFERENCES**

[1] W. Jia, Z. Cao, L. Wang, J. Fu, X. Chi, W. Gao, L.-W. Wang, The analysis of a plane wave pseudopotential density functional theory code on a GPU machine, *Comput. Phys. Commun.* **2013**, *184* (1), 9.

[2] J. P. Perdew, K. Burke, M. Ernzerhof, Generalized gradient approximation made simple, *Phys. Rev. Lett.* **1996**, *77* (18), 3865.

[3] S. Grimme, J. Antony, S. Ehrlich, H. Krieg, A consistent and accurate ab initio parametrization of density functional dispersion correction (DFT-D) for the 94 elements H-Pu, *J. Chem. Phys.* **2010**, *132* (15), 154104.

[4] A. Togo, I. Tanaka, First principles phonon calculations in materials science, *Scr. Mater.* **2015**, *108*, 1.

[5] Y. y. Wang, Z. h. Ni, T. Yu, Z. X. Shen, H. m. Wang, Y. h. Wu, W. Chen, A. T. Shen Wee, Raman studies of monolayer graphene: the substrate effect, *J. Phys. Chem. C* **2008**, *112* (29), 10637.

[6] C. Gao, M. Chang, M. Wang, H. Qu, E. Hu, Z. Kong, L. Fan, Z. Zhang, B. Wang, K. Zhai, C. Mu, Y. Cheng, Stack-related electron reconstruction induces in-plane softening in twisted bilayer graphene, *Carbon* **2025**, *243*, 120582.

[7] T. Zhang, C. Gao, D. Liu, Z. Li, H. Zhang, M. Zhu, Z. Zhang, P. Zhao, Y. Cheng, W. Huang, Pressure tunable van Hove singularities of twisted bilayer graphene, *Nano Lett.* **2022**, *22* (14), 5841.

[8] H. Mao, J.-A. Xu, P. Bell, Calibration of the ruby pressure gauge to 800 kbar under quasi‐hydrostatic conditions, *J. Geophys. Res. Solid Earth* **1986**, *91* (B5), 4673.

[9] G. J. Piermarini, S. Block, J. Barnett, R. Forman, Calibration of the pressure dependence of the R 1 ruby fluorescence line to 195 kbar, *J. Appl. Phys.* **1975**, *46* (6), 2774.
